# Response to Vitamin B12 and Folic Acid in Myalgic Encephalomyelitis and Fibromyalgia

**DOI:** 10.1371/journal.pone.0124648

**Published:** 2015-04-22

**Authors:** Björn Regland, Sara Forsmark, Lena Halaouate, Michael Matousek, Birgitta Peilot, Olof Zachrisson, Carl-Gerhard Gottfries

**Affiliations:** Gottfries Clinic, affiliated with Institute of Neuroscience and Physiology, Gothenburg University, Gothenburg, Sweden; CSIR-INSTITUTE OF GENOMICS AND INTEGRATIVE BIOLOGY, INDIA

## Abstract

**Background:**

Patients with myalgic encephalomyelitis (ME, also called chronic fatigue syndrome) may respond most favorably to frequent vitamin B12 injections, in vital combination with oral folic acid. However, there is no established algorithm for individualized optimal dosages, and rate of improvement may differ considerably between responders.

**Objective:**

To evaluate clinical data from patients with ME, with or without fibromyalgia, who had been on B12 injections at least once a week for six months and up to several years.

**Methods:**

38 patients were included in a cross-sectional survey. Based on a validated observer’s rating scale, they were divided into Good (n = 15) and Mild (n = 23) responders, and the two groups were compared from various clinical aspects.

**Results:**

Good responders had used significantly more frequent injections (p<0.03) and higher doses of B12 (p<0.03) for a longer time (p<0.0005), higher daily amounts of oral folic acid (p<0.003) in good relation with the individual MTHFR genotype, more often thyroid hormones (p<0.02), and no strong analgesics at all, while 70% of Mild responders (p<0.0005) used analgesics such as opioids, duloxetine or pregabalin on a daily basis. In addition to ME, the higher number of patients with fibromyalgia among Mild responders was bordering on significance (p<0.09). Good responders rated themselves as “very much” or “much” improved, while Mild responders rated “much” or “minimally” improved.

**Conclusions:**

Dose-response relationship and long-lasting effects of B12/folic acid support a true positive response in the studied group of patients with ME/fibromyalgia. It’s important to be alert on co-existing thyroid dysfunction, and we suspect a risk of counteracting interference between B12/folic acid and certain opioid analgesics and other drugs that have to be demethylated as part of their metabolism. These issues should be considered when controlled trials for ME and fibromyalgia are to be designed.

## Introduction

Myalgic encephalomyelitis (ME, also called chronic fatigue syndrome) and fibromyalgia (FM) are chronic clinical conditions characterized by a variety of symptoms including myalgia, fatigue and sleep disturbances. Disability to manage activities of daily life is a most common consequence of both ME and FM.

Substantial overlap exists between the two syndromes, and their similarities and differences have been much debated. They do not share a listing in the International Classification of Diseases (ICD), and they are not even classified in the same section. ME is classified as an organic neurological disorder with the code G93.3, and FM is classified in the section of Soft Tissue Disorders with the code M79.0. Both diagnoses are based on criteria and there is no specific laboratory marker. Women are affected more often than men.

**Fig 1:**
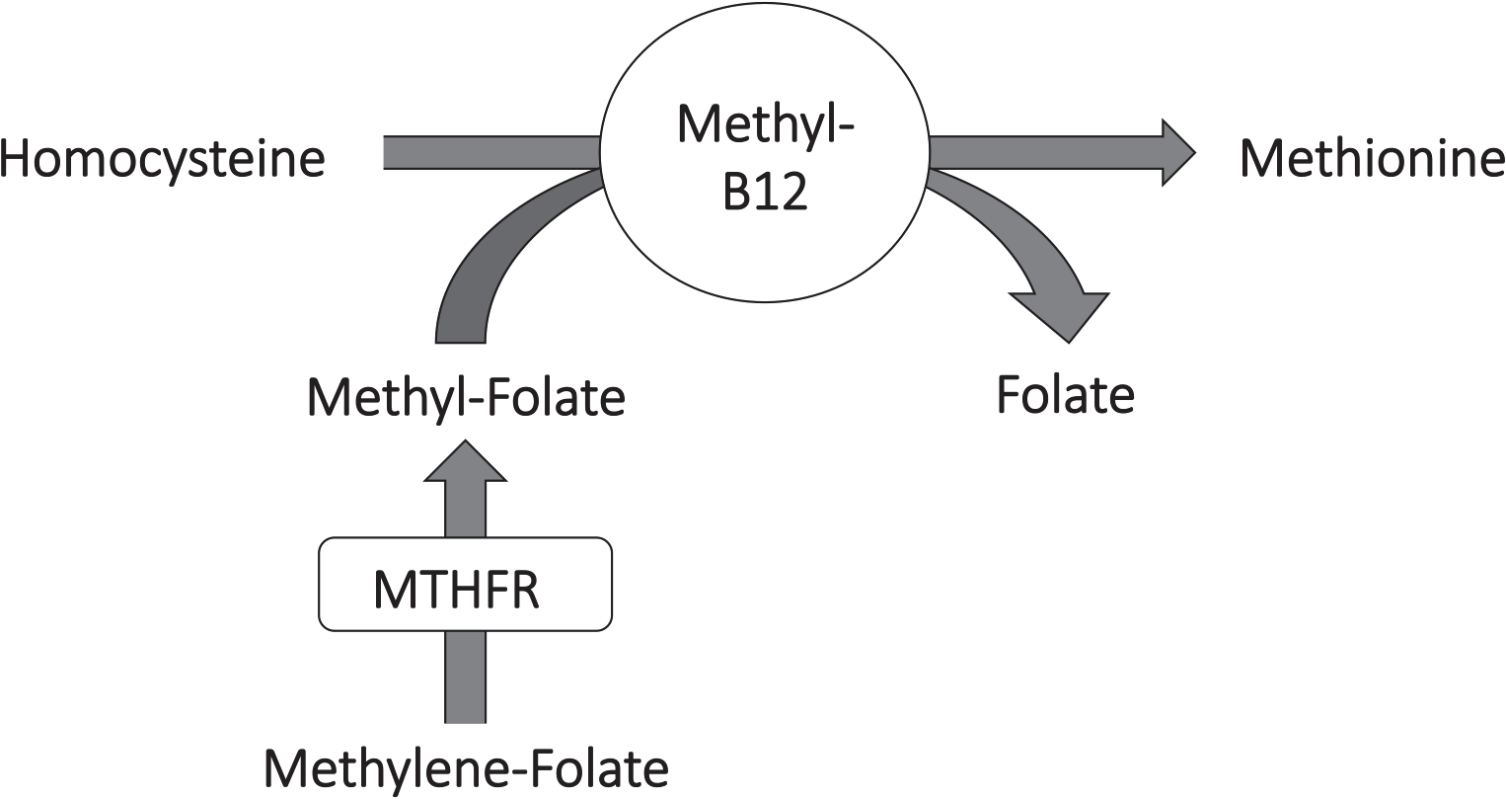
Vitamin B12 and folate in monocarbon metabolism. Schematic view of where enzyme MTHFR is contributing to synthesis of methyl-folate, and the subsequent methyl group transition from folate to B12 to homocysteine, which transforms into methionine. Activated methionine (S-Adenosyl Methionine; SAM) is the most important methyl donor in cell metabolism.

ME and FM are unexplained disorders with molecular and immunological abnormalities. In ME patients, hypomethylation is seen in a majority of certain immune cells [1] and of DNA in genes associated with immune cell regulation [2]. Although the reason for such hypomethylation can only be speculated upon, for the time being, it is interesting that the combined action of the vitamins B12 and folate (Fig 1) play a fundamental role in providing methyl groups to hundreds of substrates in various elementary cell processes. Yet another and recently revealed role of vitamin B12 is related to detoxification, by having substantial antioxidant properties [3–4]. Altogether, B12 and folate are utterly important in keeping the good health, and our paper will focus on B12/folate as a mean to improve well-being for patients with ME, with or without FM.

In 1997, we published an investigation [5] on homocysteine and vitamin B12 (cobalamin) in the cerebrospinal fluid (CSF), drawn from patients who fulfilled the criteria of both FM and chronic fatigue syndrome, the former name for ME. In comparison with a large healthy control group, all eleven patients in the study had increased homocysteine levels in CSF, although the blood levels were usually not increased. The CSF-B12 level appeared to be generally low, and CSF-homocysteine and CSF-B12 levels correlated significantly with ratings of mental fatigue. The results suggested a blockage of B12 transport over the blood brain barrier.

The different polymorphisms of MTHFR (methylenetetrahydrofolate reductase) are associated with requirements for higher doses of folic acid. As an introduction of MTHFR, see **Table 1** for an overview of expected prevalence and specific enzyme activity of its various gene variants, based on the two most commonly occurring SNPs (single nucleotide polymorphisms) described as C677T and A1298C [6–7].

**Table 1:** Genotypes of MTHFR.

Genotypes of MTHFR			Prevalence	Activity
677	1298	Name of combined genotype	%	%
CC	AA	Original genotype	16	100
CC	AC	1298C heterozygote	23	60
CC	CC	1298C homozygote	9	52
CT	AA	677T heterozygote	21	51
CT	AC	Compound heterozygote	20	36
TT	AA	677T homozygote	11	22

Overview of prevalence (ref 6) and specific enzyme activity *in vitro* (ref 7) for various genotypes of MTHFR in healthy individuals, based on combinations of the polymorphisms C677T and A1298C.

For the last fifteen years, we have been offering patients with chronic fatigue and myalgia to try out and see if they may benefit from vitamin B12 and folic acid supplements. Some patients can do well from oral treatment, but along the years we have recognized that patients with ME or FM respond best to the injective form of B12 therapy, and most of them require an injection at least once a week.

Frequent B12 injections in combination with unusually high doses of oral folic acid is not yet an established form of treatment. In recent years there was some debate on the possible risk for cancer with higher blood concentrations of folic acid. However, this issue appears to have been settled when a very large meta-analysis was recently published: Folic acid treatment of 50 000 individuals in randomized trials, with an average treatment duration of 5.2 years, had no effect on the incidence of cancer [8].

The aim of study was to compile the present status of an adequate number of patients with ME/FM, who expressed favorable response and a strong desire to continue the treatment, and to characterize such patients in more detail.

## Methods

In January-April 2014, we investigated 38 female patients with ME according to international consensus criteria [9–10]. Fifteen (40%) also fulfilled criteria for FM [11]. None of the patients had a diagnosis of pernicious anemia.

In the previous years, the included patients had reported an evident and favorable response to B12 injections in open clinical treatment. At time of inclusion in the present cross-sectional survey, all patients had been on frequent injective therapy, at least once a week, during a time period that was ranging from six months up to 20 years. In all these years, no patients were treated exactly the same since no established algorithm did exist. The individual doses of B12 and folic acid, as well as the form of B12 used (i.e. hydroxocobalamin or methylcobalamin), had been due to individual decisions made by the five doctors in interplay with their patients – to a large extent following common sense in a process of trial and error – and limited by the patient’s desire and the doctor’s permission. It was a general experience that the patients deteriorated when returning to oral treatment, or when the injection interval was prolonged. After such a dose-finding period, they continued injective B12 therapy and learned how to self-administer the injections.

In Sweden, the common form of B12 injective substrate has for more than forty years been hydroxocobalamin, provided in 1 mL ampoules with 1 mg/mL. By the end of last century, also methylcobalamin became available, in 2 mL ampoules with 5 mg/mL; i.e. an ampoule of methylcobalamin contains ten times more cobalamin than an ampoule of hydroxocobalamin. Folate in pharmacological doses is available by using tablets of folic acid (1 mg or 5 mg).

After written informed consent, the patients were included in a cross-sectional survey according to a study protocol that was approved by the Ethics Committee of Gothenburg University.

The protocol included a validated observer’s rating scale, the *Fibro Fatigue* scale (FF) [12], comprising twelve items, each rated 0–6 points. Patient’s Global Impression of Change (PGIC) was the patient’s own rating of how much the B12/folic acid treatment had helped out, i.e. minimally, much or very much improved; 1-2-3 points.

From a blood sample, the SNPs C677T and A1298C were analyzed in the gene of the enzyme MTHFR.

We registered all medications the patients were using at the time of investigation.

Adverse events were recorded at the time of inclusion in this study.

A computerized statistical program was used to calculate group differences, with Student’s t-test for independent samples, and Fischer’s exact two-tailed test for non-parametric categorical data.

## Results

At our unit, we have in the years met a few patients who experienced allergic rashes or itching due to B12 injections. However, the selected patients of this study had previously not been aware of any adverse events due to B12 and folic acid.

According to ratings with PGIC and FF, the response to treatment varied substantially between patients. Total score on FF ratings varied in the range 4–45 points; all patients below 18 were rating themselves as “very much improved” on PGIC, while most patients over 38 were “minimally improved”. In the middle zone (points 19–38), patients generally rated as “much improved”.

In order to describe the variation of status and treatments between patients, we divided them into two groups, based on the results of the total FF score. Two points or less on one item means a mild symptom, or normality. Multiplying two points with twelve items make up a sum score of 24 points, which we chose as the delimiting point of two subgroups. The 15 patients with the lowest FF scores (range 4–24) are called Good responders, and the other 23 patients are called Mild responders. Total mean score on the FF scale was 14.3±6.2 in the Good responding group and 36.4±5.5 in the Mild responding group.

The groups are compared by a number of variables in **Table 2**.

**Table 2:** Comparison of Good and Mild responders.

Variables	Good responders	Mild responders	p-value
Age (years)	51.6 ± 11.2	46.8 ± 8.9	*n*.*s*.
Duration of illness (years)	16.6 ± 5.7	13.5 ± 9.7	*n*.*s*.
Global Impression of change (pts)	2.7 ± 0.5	1.8 ± 0.6	< 0.0005
Duration of B12 injections (years)	8.1 ± 6.4	2.2 ± 2.3	< 0.0005
Interval days between B12 inj.	3.8 ± 1.9	5.8 ± 1.7	< 0.03
Folic acid mg/day	6.7 ± 6.6	1.9 ± 2.0	< 0.003
Use of high-concentrated B12	14/15 = 93%	13/23 = 57%	< 0.03
MTHFR compound heterozygote	1/15 = 7%	8/23 = 35%	*n*.*s*. (< 0.07)
Daily use of Thyroid hormone	7/15 = 47%	2/23 = 9%	< 0.02
Daily use of prescribed analgesic	0/15 = 0%	16/23 = 70%	< 0.0005
Fibromyalgia as part of ME	3/15 = 20%	12/23 = 52%	*n*.*s*. (< 0.09)

Mean ± SD or frequency/percentage for a number of variables in Good (n = 15) or Mild responders (n = 23). P-value is calculated by Student’s t-test, or by Fischer’s exact two-tailed test in the categorical data. (n.s. = no significance)

Age and duration of illness did not differ significantly between Good and Mild responders.

Duration of B12 injective therapy varied a lot, from half a year up to twenty years. Five (33%) of the Good responders and 13 (57%) of the Mild responders had started on B12 injections within the last year. B12 injective therapy had been going on for a significantly longer time in Good responders (8.1±6.4 ys) compared to Mild responders (2.2±2.3 ys).

Three of the Good responders and 12 of the Mild responders had fibromyalgia, a difference bordering to significance (p<0.09).

All but one (93%) of the Good responders were treated with methylcobalamin, while a significantly high proportion (43%) of Mild responders were using hydroxocobalamin (p<0.03). Moreover, Good responders had on average been treated with injections more frequently (interval 3.8±1.9 days) than Mild responders (interval 5.8±1.7 days). This difference was significant (p<0.03).

Oral daily dose of folic acid was significantly different (p<0.003) between Good responders (6.7±6.6 mg per day) and Mild responders (1.9±2.0 mg per day). Apart from having a higher mean dosage, the Good responders adhered to a wide range (0–20 mg per day) of individual doses, which apparently related to the individual MTHFR gene variant; three patients were homozygotes for 677T and taking 15–20 mg per day, one was compound heterozygote (i.e. 677CT and 1298AC) and taking 5 mg, four patients were heterozygotes for 677T and on average using 4.6 mg, two patients were homozygotes for 1298C and taking 2.5 and 5 mg respectively, and five patients were homozygotes for 677C and on average using 3.0 mg.

With regard to the MTHFR gene variation in the two patient groups, the most remarkable finding was the relatively high proportion of compound heterozygotes among the Mild responders. Among all patients, there were nine compound heterozygotes, eight of which were in the Mild responding group. The difference borders on significance (p<0.07).

Seven of the Good responders (47%) and only two of the Mild Responders (9%) were on substitution with thyroid hormones, which was a significant difference (p<0.02).

Opioids like tramadol, codeine and buprenorphine are regarded as strong analgesics, which is also true for duloxetine and pregabalin; the latter two are approved for the management of neuropathic pain. Daily use of such strong analgesics was significantly more frequent among Mild responders (70%), in comparison with none of the Good responders (p<0.0001). See also **Table 3**.

**Table 3:** Prescribed Analgesics.

Patient group	Tramadol	Codeine	Buprenorhine	Duloxetine	Pregabalin
Good responders n = 15	0	0	0	0	0
Mild responders n = 23	5	2	2	4	4

Number of Good and Mild responders on daily use of prescribed analgesics. Tramadol, Codeine and Buprenorphine are opioids, while Duloxetine and Pregabalin are approved for the management of neuropathic pain. One patient was using Tramadol and Duloxetine at the same time.

As should be expected by a rating scale being the basis of subgrouping, also the twelve sub-items of the FF scale differed significantly between the two groups. As one example, ‘subjective experience of infection’ was 0.3±0.7 points in the Good and 2.7±1.2 in the Mild responding group. In other words, twelve out of fifteen Good responders (80%) reported that they were no longer liable to catch infections at all, while 21 out of 23 (91%) among Mild responders reported varying degree of such liability; this difference was strongly significant (p<0.0005), according to Fischer’s exact test.

In summary, Good responders had significantly more often made use of the highly concentrated methylcobalamin preparation, which was used with yet more frequent injections, and in combination with higher daily doses of oral folic acid. Moreover, they were more often on treatment with thyroid hormones. Furthermore, none in this group was using prescribed strong analgesics, while a majority of Mild responders were using such analgesics on a daily basis.

## Discussion

We may presume that the dose-response relationship with B12 and folic acid, and the concordant ratings made by physicians (FF) and patients (PGIC), support a true positive response during a time course that was contemporary with the B12 and folic acid treatment. As experienced clinicians, we do believe that the overall positive health response cannot be a placebo effect, and that no individual would deliberately care to accept such frequent injective therapy for years, if it hadn’t been for considerable health advantages. In Good responders, the effect can be profoundly improving social life and is often long-lasting over the years. Moreover, when dosage is reduced, patients do readily deteriorate into the same almost unbearable symptoms as before.

Response to treatment is generally obvious within a few weeks from start, if the dosage is good enough. On the other hand, the longer duration of treatment amongst Good responders is partly reflecting a number of patients who had been able to adjust their dosages for several years, while half of the patients (47%) had been started only within the last year, most of them among the Mild responders, so far. Generally speaking, it takes its time to find an optimal dosage.

Surely, a number of conceivable factors may contribute to the different responsiveness between Good and Mild responders. In the following, we will bring the discussion forward by trying to answer some key questions.

### Question 1

Do the groups respond differently because they are treated differently, i.e. the Good responders are receiving more optimal treatment?

Yes, in retrospect we can see that a number of patients had not been on optimal treatment, most evidently due to low-concentrated and infrequent B12 injections, and low dosages of folic acid in relation to the MTHFR genotype. Apart from the dose, the response differences might also have been due to the form of B12 used; in a recent case report, a patient with moderate cognitive impairment responded to oral methylcobalamin but not to hydroxocobalamin [13].

Compound heterozygotes rated poorly in FF and PGIC, which may reflect that they were treated with too low dosages of folic acid; the eight Mild responders were on average using 1.75 mg per day, and the one Good responder with this genotype was taking 5 mg. As can be seen in **Table 1**, compound heterozygotes have the lowest specific enzyme activity, next to 677T homozygotes.

### Question 2

A vast majority (80%) of Good responders had only ME, while a majority (52%) of Mild responders had also FM. Maybe Good and Mild responders mainly represent separate disorders which are based on constitutively separate mechanisms, eventually causing FM or ME?

In our study, all patients fulfilled criteria for a ME syndrome, 40% of which also had the characteristics for FM. Both ME and FM are diagnoses with myalgia as a core parameter, differing in that FM patients display widespread tender points, which are extremely painful at the slightest touch. It is not clarified whether this abnormal tenderness is definitely a qualitative difference between FM and ME, or is rather a matter of variable intensity with regard to this particular aspect of the syndromes.

The basis of such tenderness was once revealed by electrophysiological experiments when succeeding pain stimuli evoked a sensitization of neurons in the dorsal horn of the spinal cord, which suggested an involvement of the NMDA (N-methyl-D-aspartate) receptor [14]. This may be of special interest because NMDA receptor dysfunction can be related to homocysteine and its metabolite (*see Question 5*). Moreover, there appears to be a growing body of evidence supporting the role for central sensitization also in ME [15].

Our Good responders did not use strong analgesics and still had much less pain than the Mild responders. Could it be that FM represents a more intense and chronic form of central sensitization in the spinal cord, while ME makes out the part of the syndrome that is especially afflicting the brain?

### Question 3

What is the role of strong analgesics for the overall response to B12 and folic acid treatment?

It is likely that a patient with FM is more prone to find relief from strong analgesics. However, many opioids, such as tramadol and codeine, have two or three methyl groups adhered to the molecule. When metabolized in the liver, these opioids are demethylated and thus activated into much stronger analgesics [16]. This may be an indication that many opioids have the ability to interact with B12/folic acid treatment, the effect of which is indeed to increase methylation. Such a notion may find support by two FM patients in our study, who were using tramadol as analgesic. After the examination, they were allowed to intensify B12 and folic acid, and both of them responded most negatively: They experienced intensified generalized pain, “it was like the effect of tramadol disappeared”.

Duloxetine (SNRI) is also undergoing demethylation as part of its normal metabolism, which makes it potentially more apt to interact with B12/folic acid treatment in pharmacological dosages. We have made repeated clinical observations that B12/folic acid may cause paradoxical sedation when combined with SSRI/SNRI drugs; the sedation vanishes when the SSRI/SNRI dosage is reduced or cancelled. This might be in analogy with the old pharmacological observation that demethylation converts potent sedatives into antidepressant agents [17]. Maybe B12/folic acid, by its remethylating potential, reverses the normal demethylation of SSRI/SNRI and thus keeps SSRI/SNRI in its original form, which is much more sedating than its demethylated form?

Good responders were apparently receiving more optimal dosages of B12 and folic acid, and they could do without the probably interfering effects of strong analgesics. Maybe Mild responders were worse off because they were ‘trapped’ by the analgesic drugs and thus could not tolerate additional and intense B12/folic acid treatment?

### Question 4

Why is treatment with thyroid hormones significantly more common among Good responders?

Nine patients were using thyroid hormones because of hypothyroidism, and their treatment was not initiated at our unit. In addition, three patients had been on substitution during pregnancy. Seven of the Good responders had positive titers of thyroid antibodies (47%).

Thyroid antibodies are common among middle-aged women [18], but the fact that Good responders were significantly more often on thyroid hormone substitution may alert us to the possibility that patients with ME/FM are in the risk zone of having a thyroid imbalance. When needed, thyroid treatment may contribute to the overall treatment effect, i.e. in combination with B12 and folic acid.

The obvious conclusion is that patients with ME/FM should always be tested for thyroid function.

### Question 5

What is the reasonable mechanism behind a positive response to B12/folic acid treatment?

Classical B12 deficiency (*pernicious anemia*) is most often explained on the basis of gastrointestinal autoimmunity against gastric parietal cells or intrinsic factor, which results in malabsorption of B12 at the gut level [19]. The initial blood level of B12 should be very low in pernicious anemia. After an initial series of frequent intramuscular injections of hydroxocobalamin for a week or two, the usual maintenance dose is an injection once a month, or less frequent, or a daily oral dose (1mg) of cyanocobalamin.

Based on a recent study of ME patients at our unit, results from analysis of HSP60 epitopes were compatible with the presence of infection-induced autoantibodies [20]. Thus, data are accumulating that ME may have autoimmune features as part of its makeup.

As mentioned in the introduction, we have once shown that patients who fulfilled criteria of both ME and FM had increased homocysteine levels in the cerebrospinal fluid [5]. Homocysteine [21] and its metabolite [22] have neurotoxic potentials at the NMDA receptor (*see Question 2*), which enroll them as candidate triggers of the spinal cord sensitization in FM, and a possible cause of neuropathic pain. On the other hand, increased homocysteine levels can be normalized by the concerted action of B12 and folate, but do we know how to accomplish enough concentrations of B12 and folate across the blood brain barrier? Maybe part of the answer is to considerably increase the concentrations of B12 and folate in the blood?

Regarding the patients in focus of the present study, a relevant issue is whether there are any suspect targets at the level of the blood brain barrier, a dysfunction of which may explain why highly increased concentrations of B12 and folic acid are required? We suggest that the megalin receptor in the choroid plexus can be of special interest. Megalin is expressed in the choroid plexus [23], and dysfunction of the megalin receptor affects the transport over barriers, not only of vitamin B12 [24], but also of folate [25] and transthyretin, i.e. the transporter of thyroxine [26]. In close connection with the blood stream, the function of a receptor can be reduced for various reasons, such as by blocking or binding autoantibodies, or by toxic destruction from heavy metals, like cadmium or mercury [27–28].

### Conclusions

Frequent injections of high-concentrated vitamin B12, combined with an individual daily dose of oral folic acid, may provide blood saturations high enough to be a remedy for good and safe relief in a subgroup of patients with ME/FM. Moreover, we suspect a counteracting interference between B12/folic acid and certain opioid analgesics and other drugs which have to be demethylated as part of their metabolism. Furthermore, it is important to be alert on co-existing thyroid dysfunction. These issues should be considered when controlled trials for ME and fibromyalgia are to be designed.
